# The magnitude of the partial volume effect in SPECT imaging of the kidneys: a phantom study

**DOI:** 10.1186/s40658-022-00446-2

**Published:** 2022-03-14

**Authors:** Andreas Grings, Camille Jobic, Torsten Kuwert, Philipp Ritt

**Affiliations:** Clinic of Nuclear Medicine, University Hospital Erlangen, Erlangen, Germany

**Keywords:** Kidney, Phantom, Recovery coefficient, 3D-print, SPECT, CT, ^99m^Tc

## Abstract

**Background:**

Single-photon emission computed tomography (SPECT) can cause an over- or underestimation of tissue activity concentration due to limitations in spatial resolution compared to the structures under study. This is commonly referred to as partial volume effect (PVE). Ideally, the PVE should be controlled for and corrected. One such correction method involves determining recovery coefficients (RC) from phantom measurements. In the literature, several studies applying simplified geometries are available. In this study, we aimed to determine kidney PVE for realistic kidney geometries. Furthermore, we proposed a new surrogate metric for predicting the extent of PVE in kidneys.

**Material and methods:**

Based on patients’ CT data, we manufactured fillable phantoms using a 3D-printer. Nine cortex-only and ten whole-parenchyma phantoms were obtained, and one ellipsoidal phantom for comparison. To measure PVE, we placed the phantoms in a torso phantom and filled them with a specified activity concentration. The phantoms’ RCs were determined from fully quantitative SPECT/CT acquisitions at three different target-to-background ratios (TBRs). Additionally, the surface area-to-volume (SA:V) ratio was determined for all phantoms and correlated with RCs.

**Results:**

For SPECT reconstructions with 36 iterations, average RC ± one standard deviation at a 10-to-1 TBR was 76.3 ± 1.5% and 48.4 ± 8.3% for whole-parenchyma and cortex-only phantoms, respectively. The RC for the ellipsoidal phantom was 85.4%. The RC for whole-parenchyma was significantly higher than for cortex-only phantoms (*p* < 0.01). The RC variance was significantly higher for cortex-only phantoms (*p* < 0.01). A highly significant correlation of the SA:V ratio and RC was found for all phantoms. (*R*^2^ of linear regression was between 0.96 and 0.98.)

**Conclusion:**

Changes in the specific shape of the kidneys cause large changes in PVE magnitude. Therefore, RCs derived from more simple phantoms are most likely insufficient to correct the PVE in patient images. Furthermore, one should account for the fact that intra-renal activity distribution significantly influences the extent of PVE. Additionally, we found that the SA:V ratio excellently models kidney RCs; potentially, this approach could also be applied to other geometries and represents an alternative to full imaging process simulations to determine the extent of PVE.

## Background

The partial volume effect (PVE) confounds the measurement of activity concentration in emission imaging, such as in Single Photon Emission Computed Tomography (SPECT) and Positron Emission Tomography (PET) [1]. The PVE is caused by the limited spatial resolution of these imaging systems compared to organ size. In SPECT systems, spatial resolution is reduced primarily by collimator performance. SPECT collimators are typically designed with a tradeoff between spatial resolution and detection efficiency; typically, spatial resolution cannot be increased without decreasing detection efficiency. Besides the collimator design and geometry, spatial resolution is also influenced by the intrinsic resolution of the detector.

Volume of interest (VOI) in structures with heterogeneous activity distribution that are smaller than three times the full width at half-maximum (FWHM) of the spatial resolution or that are located at the edge of even larger structures is affected by PVE [2]. Their activity is under or overestimated to a varying degree, depending on the combination of spill-in and spill-out effects. Spill-in effects result from increased activity outside the VOI or structure. The activity is integrated into the VOI because of the limited spatial resolution. In this case, the intensity inside the VOI appears increased. Spill-out effects result from the activity of the VOI/structure distributed over the borders of the VOIs (again due to the limited spatial resolution), causing a loss of the signal in the VOI; the intensity inside the VOI is decreased. The ratio between image-derived- and true activity is commonly termed the recovery coefficient (RC). The degree of the PVE and thus the RC depend on the spatially varying system resolution of the imaging system, the patient (e.g. motion), and the true radioactivity distribution in the image. Several publications have reported on the dependency of RC, object size, contrast ratio, and spatial resolution, e.g. [3–5].

Clinical SPECT systems apply parallel-hole collimation and offer FWHMs between 7 and 15 mm when ^99m^Tc is used. For ^177^Lu, SPECT spatial resolution is lower at source-to-collimator distances used in clinical practice. [6]

The PVE not only affects visual SPECT and PET image evaluation, but also the determination of radionuclide uptake in absolute units as necessary, e.g. for dosimetry of radionuclide therapy.

PVE correction is the ultimate goal. However, no generally accepted correction method exists and most correction algorithms proposed are too complex for routine use in clinical practice. As an alternative, some researchers have recommend using experimentally derived RCs [6].

Kidneys are specifically targeted for assessment in dosimetry for ^177^Lu therapy since kidneys are often regarded as dose-limiting [7]. Consequently, determining the extent of kidney PVE and correcting the PVE with RCs is important for obtaining accurate dosimetry results.

Several studies have reported on 3D printed phantoms for use in PET and SPECT imaging. A recent review on the topic can be found in [8]. In the following, we provide details for studies that we consider relevant for our work.

Woliner-van der Weg et al. generated 3D printed anatomic pancreas and kidney phantoms based on magnetic resonance images (MRI) from one patient, aiming at optimizing the SPECT/CT reconstruction settings of beta cell imaging [9]. They concluded that the results of 3D printed phantoms resembled that of clinical patient studies and had similar artefacts. They also evaluated SPECT/CT-derived determination of several quantitative metrics and found that the activity concentration of the pancreas was underestimated relative to that of the kidneys. In another recent study, Kühnel et al. 3D printed fillable patient-specific thyroid replicas, which were subsequently used for gamma probe calibrations, and concluded that the creation of patient-specific organ phantoms based on 3D printing and the determination of PVE are feasible [10]. Tran-Gia et al. designed and manufactured 3D printed, one- and two-compartment (cortex and medulla) kidney phantoms and determined the extent of PVE in ^177^Lu SPECT imaging. These phantoms resembled two hollow ellipsoids that represented the walls of the medulla and cortical compartments. That study revealed that quantitative SPECT/CT-derived activity distribution differed from the actual distribution in the phantom [11] and concluded that the bias introduced by PVE was so extensive that it needed correction. In our view, one limitation of this study is that these phantoms resembled simplified kidney structures but failed to account for patient-specific differences and specifically the highly complex boundary between renal medulla and cortex.

To the best of our knowledge, no studies have determined RCs for multiple patient-specific kidney geometries and different intra-renal activity distributions. Consequently, this study aimed to evaluate the magnitude of PVE in patient-specific kidney geometries at two different intra-renal activity distributions and with three different kidney-to-background contrasts.

## Methods

**Fig. 1:**
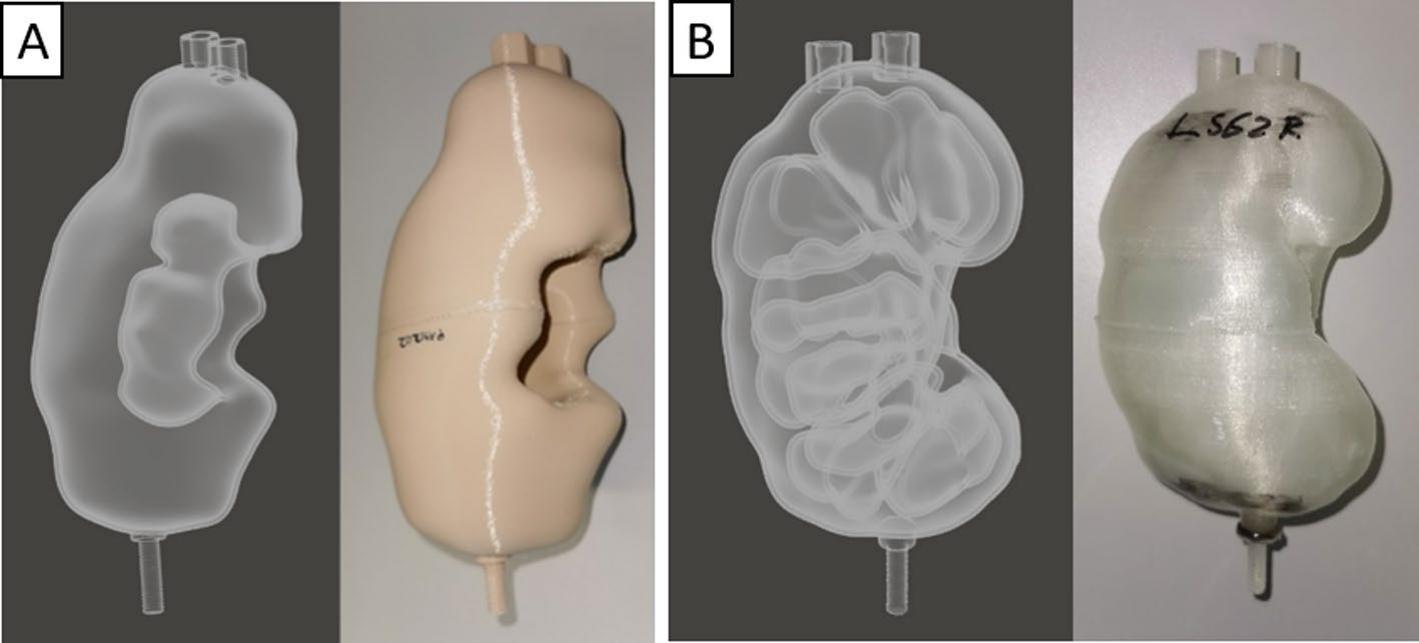
Computer-aided-design models and printed phantoms. Computer-aided-design (CAD) models (left side) and printed phantoms (right side) for whole-parenchyma (**A**) and cortex-only phantoms (**B**)

To determine the PVE, 19 kidney phantoms were manufactured. All phantoms were designed to have one single compartment, either encompassing the whole-parenchyma (medulla + cortex) or cortex only. We wanted the former to resemble a situation in which a radiopharmaceutical drug is excreted without specifically binding to any part of the kidney and the latter to resemble a radiopharmaceutical drug that binds to the cortex of the kidney. Since both phantom types were complex compared with simple geometric forms, we also added one ellipsoid phantom with a volume similar to that of a kidney. Examples of the kidney CT models and printed phantoms are provided in Fig. 1.

The kidney phantoms were created based on segmented CT data of 11 randomly selected patients (women and men) referred to the Clinic of Nuclear Medicine at the University Hospital Erlangen for radionuclide therapy with [^177^Lu]Lu-DOTA-octreotide or PET/CT with [^18^F]FDG. The institutional review board (IRB or equivalent) approved this study, and all subjects signed a written informed consent.

We acquired CT data for the whole-parenchyma phantoms on a Siemens Symbia T2 system (Siemens Healthineers, Germany), as part of a post-therapeutic SPECT/CT acquisition. The CT parameters were as follows: tube voltage 130 kV; tube time–current 30 mAs reference; slice collimation 2 × 2.5 mm; and pitch 1.8. CT images were reconstructed using the manufacturer’s implementation of the filtered-back-projection algorithm and a B31s kernel at a slice thickness of 3 mm.

CT data for the cortex phantoms were acquired on a Siemens Biograph mCT 40 system (Siemens Healthineers, Germany) as part of a regular PET/CT acquisition with intravenous X-ray contrast. All datasets were obtained in the arterial phase to enable differentiation between the medulla and cortex. Acquisition parameters were as follows: Tube voltage 120 kV; tube time–current 133mAs reference; slice collimation 16 × 1.2 mm; and pitch 1. Here, images were reconstructed again using the manufacturer’s implementation of filtered-back-projection and a B30f kernel at a slice thickness of 1.5 mm.

Together, 10 left and 9 right kidneys from 11 patients were selected for further processing; 10 kidneys for whole-parenchyma phantoms and 9 kidneys for cortex-only phantoms.

### Segmentation and design of kidney phantoms

**Fig. 2:**
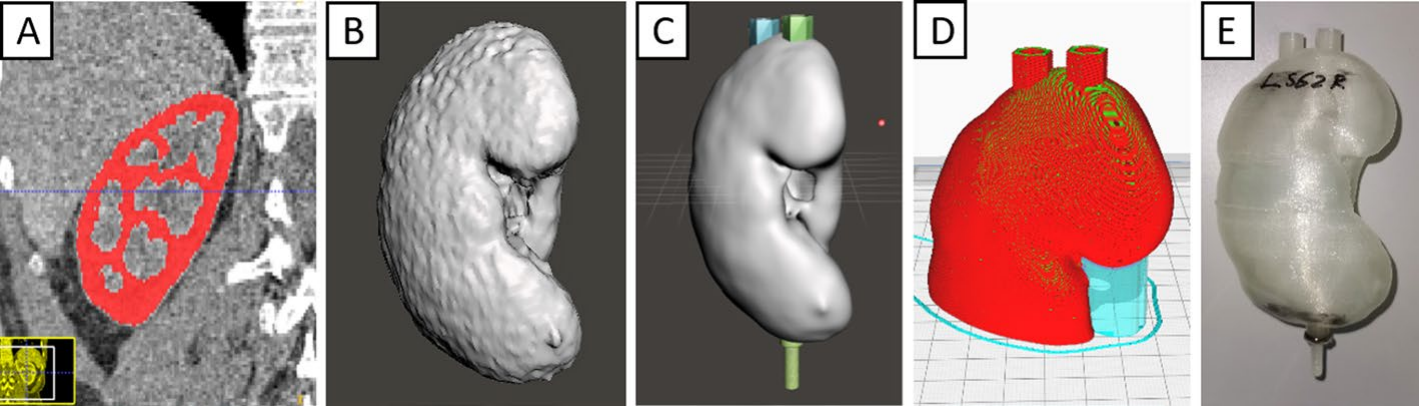
Graphical representation of the workflow for creating kidney phantoms. Workflow for creating a kidney phantom. **A** Segmentation, **B** segmented model, **C** modified model, **D** half phantom for printing, **E** kidney phantom

A short overview of the processing steps is provided in Fig. 2. The kidneys were segmented from patient CT data using the ITK-SNAP program (www.itk-snap.org, Version 3.8.0) [12]. For this purpose, the respective structure outlines were hand-contoured in transversal slices of the CT images. This segmentation was then exported as a surface model and imported to the computer-aided-design (CAD) program, Meshmixer (Autodesk, Inc.; Version 3.5.474).

Within the CAD program, the kidney models were smoothed to evade a rough surface which could otherwise negatively impact the printing process. Additionally, the smoothing implied a lower size limit to the surface-defining features, which could otherwise lead to excessively large measured surfaces and also lessens the impact of CT scanning and reconstruction parameters onto the surface size. We took the Meshmixer (“Smooth”) tool to proceed the smoothing. This tool has some parameters to influence the smoothing of the model. First of all, we have chosen the Smoothing Type “Shape Preserving”, and this will lead to a surface model where the shape of the triangles is close to the original model. Second, we took a Smoothing Scale of 20, and this parameter controls the impact of the smoothing, where 0 would be no smoothing and 100 maximal possible. Also, we used the standard Meshmixer Smoothing value of 1, and a lower value would influence the smoothing within the scale and would lead to artefacts and Constraint Rings of 3, which has no influence to our model, because we smoothed the whole model and this would only affect the borders to the outside of the smoothing. A wall with 1.2 mm thickness was placed around the segmentation so that a cavity corresponding to the kidney was created. After this, a model of a screw was integrated at the phantom’s bottom (corresponding to the direction oriented towards a patient’s feet) to attach it to the threads in an abdomen-mimicking phantom. To fill the phantoms, two access ports were integrated on their top.

### Determination of SA:V

The ratio of surface area-to-volume of phantoms (SA:V) was determined from the initial segmentation of CT images, which was imported in the CAD application and post-processed as indicated above. The used CAD program offers a sub-module (“stability” module) for calculating surface area and volume of models, which we used for determining these parameters. The same steps would be carried out for patient images. The given spatial resolution and voxel size of the CT images and segmentations, as well as the smoothing of the model within the CAD program potentially, influence the measured surface-area. A ‘higher’ (resolving smaller details) spatial resolution and smaller smoothing could potentially cause a continuous increase of the surface.

### Kidney phantom printing

The resulting models were printed using the Ultimaker 3 Extended 3D printer (Ultimaker B.V., Utrecht, Netherlands), a fused deposition modelling (FDM) printer capable of dual-extrusion. Polylactic acid (PLA) was used to print the phantom walls, and polyvinyl alcohol (PVA) was used as a water-soluble support. To reduce print times and the amount of required support material, all kidney models were divided into two halves, as shown in Fig. 2D. After printing, the PVA support structures were removed by repeated rinsing with water. After drying, the matching halves were glued together. If necessary, phantoms were sealed by coating with epoxy resin to prevent leakage of the filled phantom.

### SPECT/CT measurement and evaluation

Due to the frequent and necessary phantom handling (filling phantoms over sixty times), we decided to perform the experiments with ^99m^Tc instead of ^177^Lu for radiation safety reasons. Taking into account differences in detection sensitivities and branching ratios of the two radionuclides, we aimed to fill the phantoms with ^99m^Tc with approximately 100 kBq/ml and an acquisition time of 7.5 min, which we surmised would result in a kidney count density similar to a standard post-therapeutic patient acquisition, which we determined to have a ^177^Lu activity concentration of 420 kBq/mL and an acquisition time of 15 min. For the actual experiments, phantoms were placed two at a time in a torso background phantom (equivalent to NEMA-NU 2007 body phantom, PTW-Freiburg, Freiburg, Germany). Three different sets of measurements were made with various target-to-background ratios (TBRs).

- In the first set, the kidney phantoms were filled to a concentration of 102.5 kBq/ml ^99m^Tc, and the background remained cold (no activity), which we referred to as the infinity TBR.
- In the second set, kidney phantoms were filled to a concentration of 100.7 kBq/mL ^99m^Tc, and the backgrounds were filled to a concentration of 10.4 kBq/ml ^99m^Tc, which we referred to as the 10:1 TBR (actual ratio 9.7:1).
- In the third set, kidney phantoms and background concentrations were filled to concentrations of 98.44 kBq/ml and 20.15 kBq/mL ^99m^Tc, respectively. We referred to this as the 5:1 TBR (actual ratio 4.9:1)

To facilitate reconstructed SPECT/CT image evaluations/segmentations, CT contrast medium (Iomeron 350, Bracco Imaging, Konstanz, Germany) was added to the kidney phantoms at a ratio of 1:100, which shifted the Hounsfield (HU) values of this compartment slightly to approximately 70 HU.

All phantom acquisitions were carried out on a clinical SPECT/CT device (Symbia Intevo Bold, Siemens Healthineers, Germany) using LEHR collimation, a 180° detector configuration, 120 projections over 360° (3° sampling) acquired in 60 views at 7.5 s, a 256 × 256 projection matrix size, a pixel spacing of 2.40 × 2.40 mm, an Energy window of 130.5 to 151.6 keV, and a lower scatter window from 109.3 to 130.5 keV. To obtain quantitative values, the SPECT system was calibrated using Co-57 precision reference sources as part of Siemens xSPECT Quant technology (Siemens). The CT parameters of the hybrid SPECT/CT acquisition were as follows: tube voltage 130 kV; tube time–current 25 mAs reference; Siemens Care Dose 4D tube current modulation; slice collimation 16 × 1.2 mm; and pitch 1.5. CT images were reconstructed using the manufacturer’s implementation of filtered-back-projection and the B31s kernel at a slice thickness of 3 mm.

SPECT projection data were reconstructed using Siemens xSPECT Quant technology, based on an ordered-subset conjugate-gradient (OSCG) algorithm. Corrections were applied based on the manufacturer’s implementations. In brief, scattered radiation was corrected using a dual-energy window method and attenuation correction was CT-based, with a pixel spacing of 1.95 × 1.95 mm, a matrix of 256 × 256, 201 slices, and a slice thickness of 1.95 mm. Two reconstructions were carried out, one with 36 iterations and one with 72 iterations both with 1 subset, resulting in fully quantitative SPECT data, with voxel units in kBq/ml. The RC for each of the kidney phantoms was determined from these reconstructions by semi-automated segmentation of the respective volumes of interest (VOIs) on a Siemens Symbia.net workstation, using the *Volumetric Analysis* program as described below.

**Fig. 3:**
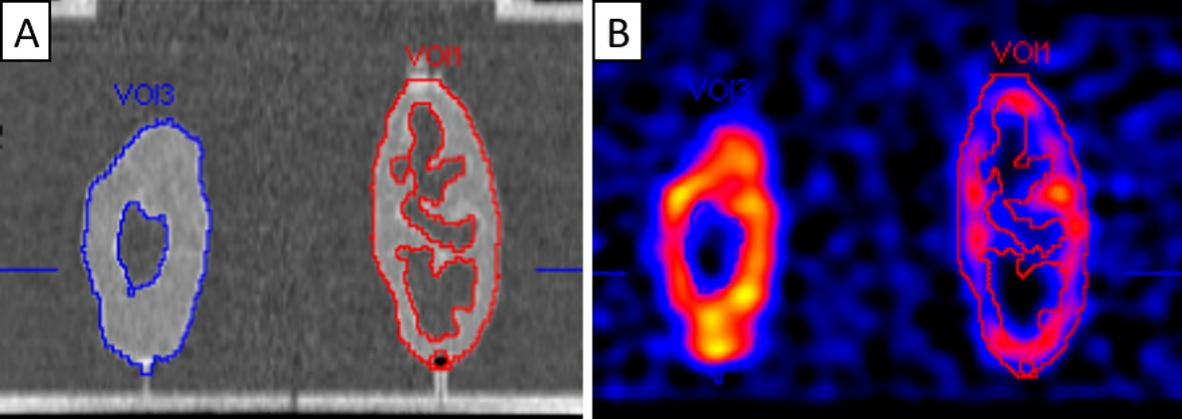
SPECT/CT images of kidney phantoms. SPECT/CT images of the phantoms Pat10L (whole-parenchyma) and Pat1R (cortex only) acquisitions. The phantoms were filled with a 10:1 target-to-background ratio. The volume of interest (VOI) was defined as the isocontour on the CT image (**A**) in such a way that all voxels inside the phantom were included. The threshold of the isocontour was adjusted so that the volume of the VOI was as close as possible to the actual filling volume of the phantoms. The thresholds for VOI1 and VOI3 were 88%. The VOI was subsequently transferred to the SPECT image (**B**) to determine the recovery coefficient of the phantom

For each kidney phantom, we aimed to create a CT-defined VOI enclosing all voxels that represented radioactive liquid within the phantom. For this, we defined the position and size of an isocontour in such a way that all voxels inside the phantom were included. We visually adjusted parameters so that the volume of the resulting VOI was as close as possible to the fillable volume of the phantom. The VOIs were then transferred to reconstructed SPECT data, and the average concentrations of the VOIs were determined. Finally, the RC of each phantom was calculated as the ratio of SPECT-determined concentrations to the actual concentrations as defined from the phantom filling procedure (Fig. 3).

### Evaluation and statistical testing

For evaluating whether the RCs were significantly different between whole parenchyma and cortex-only phantom types, we used the nonparametric Mann–Whitney U test. Additionally, we applied the Levene test to determine if RC values belonging to a certain phantom type have higher variances.

We applied a nonparametric Wilcoxon signed-rank test to determine whether the RCs of phantoms filled at different TBRs were statistically different. Similarly, we used the nonparametric Wilcoxon signed-rank test to analyse whether the number of iterations (36 vs. 72) led to significantly different RCs.

To determine RC dependency on the SA:V ratio, we applied a standard least-squares regression analysis, using a linear model with equal weights for all data points (GraphPad Prism, Version 8.3, GraphPad Software LLC).

## Results

### Size of phantoms

The 19 kidney phantoms differed in size and shape. The cortex-only phantoms had an average volume of 123.3 cm^3^, which was less than the average volume of the whole-parenchyma phantoms of 171.2 cm^3^. Yet, the cortex-only phantoms had a larger surface area (average 370.7 cm^2^) compared with the whole-parenchyma phantoms (243.8 cm^2^) due to the higher complexity of their shape. Subsequently, the SA:V ratio was different for the two types of kidney phantoms. The SA:V ratio in the cortex-only and whole-parenchyma phantoms averaged 3.0 cm^−1^ and 1.4 cm^−1^, respectively. The elliptical reference phantom had a volume of 212.5 cm^3^, a surface area of 180.2 cm^2^, and a SA:V ratio of 0.8 cm^−1^. Table 1 lists the volume, surface area, and SA:V ratio for each phantom.

**Table 1:** Volume, surface area, and surface area-to-volume (SA:V) ratio for all kidney phantoms in the study

Cortex-only phantom	Volume [cm^3^]	Surface area [cm^2^]	SA:V [cm^−1^]	Whole-parenchyma phantom	Volume [cm^3^]	Surface area [cm^2^]	SA:V [cm^−1^]
Pat1L	135.5	367.3	2.7	Pat7L	160.1	230.9	1.4
Pat1R	129.3	410.3	3.2	Pat7R	167.9	246.6	1.5
Pat2L	124.4	398.6	3.2	Pat8L	182.0	256.8	1.4
Pat2R	94.8	365.9	3.9	Pat8R	181.8	257.7	1.4
Pat3L	70.4	244.4	3.5	Pat9L	167.2	229.8	1.4
Pat3R	156.3	376.6	2.4	Pat9R	187.9	264.1	1.4
Pat4L	162.0	419.5	2.6	Pat10L	173.1	243.7	1.4
Pat5R	113.8	388.6	3.4	Pat10R	171.1	260.1	1.5
Pat6L	123.0	365.2	3.0	Pat11L	168.6	234.9	1.4
				Pat11R	151.9	213.8	1.4
AVG ± SD	123.3 ± 26.8	370.7 ± 48.4	3.1 ± 0.4		171.2 ± 10.2	243.8 ± 15.5	1.4 ± 0.0
Ellipsoid	212.5	180.2	0.8				

The last line lists the ellipsoidal phantom for comparison

### Recovery coefficients

**Fig. 4:**
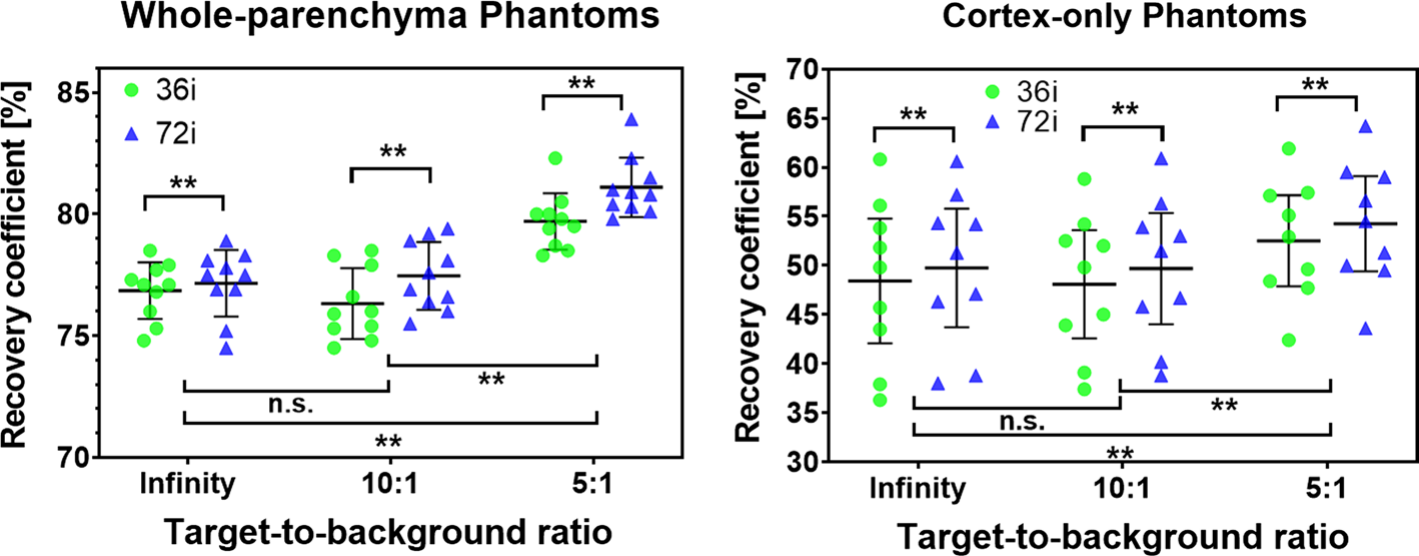
Measured recovery coefficients of each phantom. Measured recovery coefficients (RCs) of each phantom in the study for any combination of the target-to-background ratio and iteration number (green points, 36 iterations; blue triangles, 72 iterations). Values for the whole-parenchyma and cortex-only kidney phantoms are shown in left and right subfigures, respectively. The solid horizontal line for each group represents the mean; the associated whiskers indicate plus/minus one standard deviation. Additionally, the result of the nonparametric testing is provided. Two asterisks (**) indicate a statistically significant difference with a *p* < 0.01, not significant (n.s.) indicates that a statistically significant difference was not found. Test results between the RCs of reconstructions with different iteration numbers are displayed above data points. Test results between RCs at different TBRs are shown below the data points. Values were obtained for LEHR collimation and the radionuclide ^99m^Tc

The measured recovery coefficient for LEHR collimation and the radionuclide ^99m^Tc for each phantom at any TBR combination and iteration number are provided in Table 2 (cortex-only) and in Table 3 (whole-parenchyma). A graphical representation is given in Fig. 4.

**Table 2:** Recovery coefficients (RCs) for the cortex-only kidney phantoms, three different target-to-background ratios, and two different SPECT reconstruction iteration numbers

Cortex-only phantom name	Recovery coefficients [%] 36 iterations			Recovery coefficients [%] 72 iterations		
	Target-to-background ratio			Target-to-background ratio		
	Infinity	10:1	5:1	Infinity	10:1	5:1
Pat1L	56.1	54.2	57.4	57.2	56.3	59.5
Pat1R	60.8	58.8	61.9	60.6	60.9	64.2
Pat2L	45.7	45.0	49.6	47.1	46.7	51.3
Pat2R	43.5	43.9	47.7	46.3	45.8	50.0
Pat3L	37.9	37.4	42.4	38.8	38.8	43.6
Pat3R	36.3	39.1	48.4	38.0	40.2	49.5
Pat4L	53.8	52.5	57.1	54.3	53.9	59.0
Pat5R	51.8	52.0	55.1	54.2	53.0	56.6
Pat6L	49.8	49.8	52.9	51.3	51.5	54.5
AVG ± SD	48.4 ± 8.3	48.0 ± 7.2	52.5 ± 6.0	49.8 ± 7.9	49.7 ± 7.4	54.2 ± 6.3

The AVG ± SD row denotes the averages plus/minus one standard deviation of all cortex-only phantom RCs. Values were obtained for LEHR collimation and the radionuclide ^99m^Tc

**Table 3:** Recovery coefficients (RCs) for the whole-parenchyma kidney phantoms, three different target-to-background ratios, and two different SPECT reconstruction iteration numbers

Whole-parenchyma phantom name	Recovery coefficients [%] 36 iterations			Recovery coefficients [%] 72 iterations		
	Target-to-background ratio			Target-to-background ratio		
	Infinity	10:1	5:1	Infinity	10:1	5:1
Pat7L	75.3	74.5	78.5	75.2	75.5	80.3
Pat7R	76.0	75.3	80.5	76.9	76.6	82.3
Pat8L	77.3	75.9	78.3	77.5	77.6	80.4
Pat8R	76.8	76.6	80.0	76.9	78.1	80.1
Pat9L	77.9	74.8	79.4	78.9	76.0	80.8
Pat9R	74.8	75.4	78.7	74.5	76.4	79.8
Pat10L	78.5	78.5	82.3	78.3	79.4	83.9
Pat10R	77.1	76.0	79.5	77.8	76.9	81.0
Pat11L	77.1	78.3	80.0	77.5	79.2	81.5
Pat11R	77.7	77.9	79.8	78.1	78.9	80.9
AVG ± SD	76.9 ± 1.2	76.3 ± 1.5	79.7 ± 1.2	77.1 ± 1.4	77.5 ± 1.4	81.1 ± 1.2
Ellipsoid	85.4	82.8	89.0	85.4	83.3	89.6

The AVG ± SD row denotes the average plus/minus one standard deviation of all whole-parenchyma phantoms RCs. The last line contains the RCs of the ellipsoidal phantom for comparison. Values were obtained for LEHR collimation and the radionuclide ^99m^Tc

We found that a higher number of iterations resulted in a significantly higher RC (*p* < 0.01) by an average of 1.2 percentage points (pp) for 72 iterations. For clarity, in this study, we report the regression for 72 iterations only; results for 36 iterations led to the same interpretation and conclusion.

When comparing the RCs for the different TBR ratios, the values between the infinity and 10:1 ratio did not significantly differ. The RCs for the 5:1 TBRs were significantly higher (*p* < 0.01) than the infinity and 10:1 TBRs, averaging 4.2 pp and 4.1 pp higher, respectively.

The RCs for whole-parenchyma phantoms were significantly higher (*p* < 0.01) compared with cortex-only phantoms, averaging 27.4 pp., 27.8 pp., and 26.8 pp higher for the infinity, 10:1 and 5:1 TBRs, respectively. Additionally, the variance of RCs for the cortex-only phantoms was significantly higher (*p* < 0.01) than that for the whole-parenchyma phantoms. For example, the standard deviation (SD, reported for consistency with other studies) at the 10:1 TBR was 6.0 pp higher for the cortex-only phantom than the whole-parenchyma phantoms (SD of 7.4% vs. 1.4%).

### Regression between the RCs and SA:V ratio

**Fig. 5:**
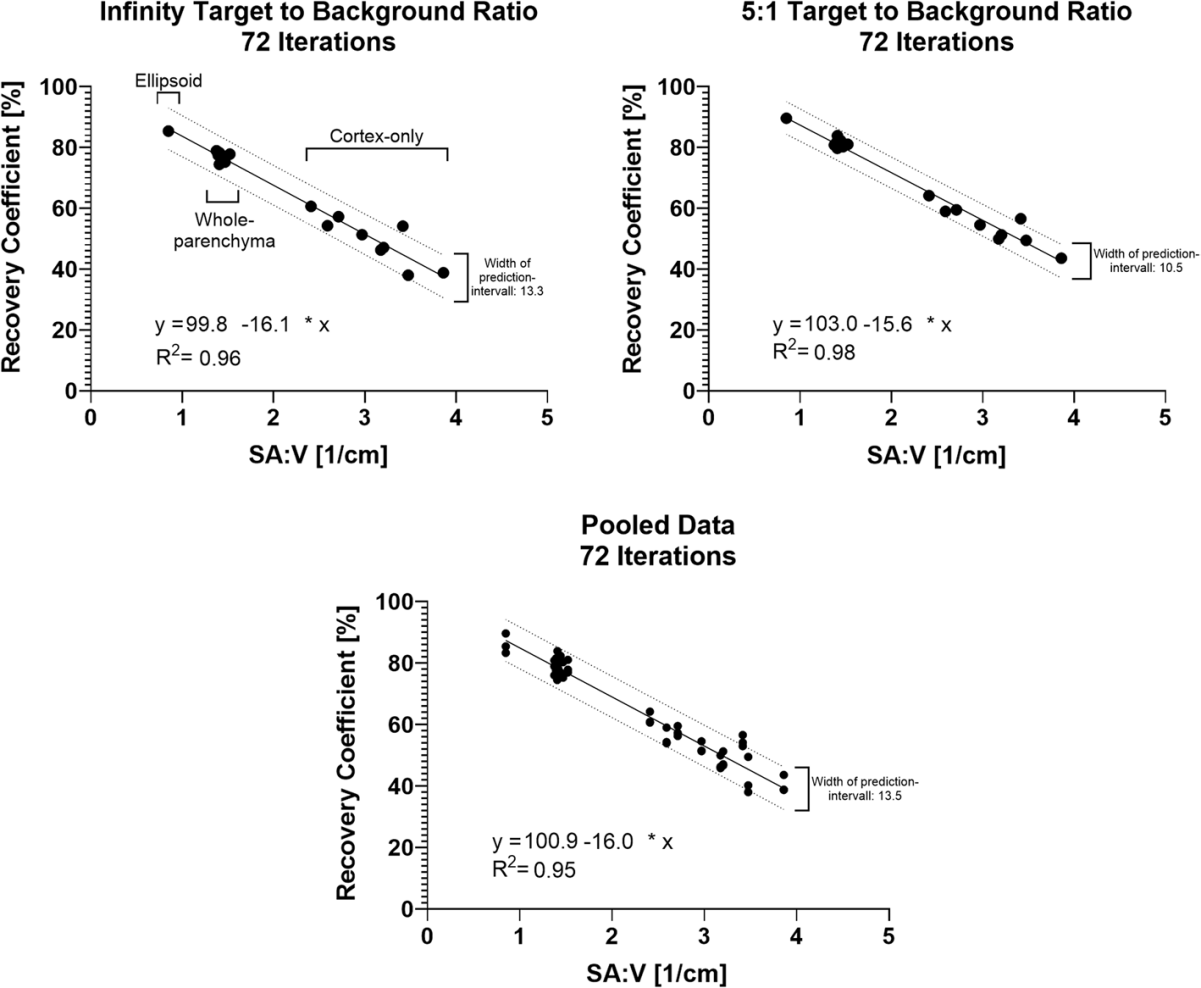
Correlation of recovery coefficients and surface-area-to-volume ratio. Recovery coefficients vs. surface-area-to-volume (SA:V) ratios for all phantoms in the study. The infinity (cold background) (top left graph), 5:1 target-to-background ratios (top right), and the pooled data (bottom, infinity, 5:1 and 10:1 TBR) are shown with 72 iterations. Additionally, the best fit (solid line), the 95% prediction intervals (dashed lines) of the linear regression analysis, the average width of the prediction intervals, the equation of the best fit, and the goodness of fit (given as R-squared) are provided. The additional labels in the top left image indicate the position of the phantom type (ellipsoid. whole-parenchyma, cortex-only) in the graph. Values were obtained for LEHR collimation and the radionuclide ^99m^Tc

The regression analysis between the RCs and SA:V ratios of the phantoms led to linear fits with R-squared values of 0.96, 0.97, 0.98, and 0.95 for the infinity, 10:1, 5:1, and pooled TBRs, respectively. The corresponding optimal fit parameters are provided in Table 4. A graphical representation of the regression analysis is given in Fig. 5 for the infinity, 5:1, and pooled TBRs. The analysis and curve for the 10:1 TBR were similar to those of the infinity TBR. All results represent the regression between RCs and SA:V ratio for the radionuclide ^99m^Tc and LEHR collimator on a Siemens Intevo Bold.

**Table 4:** Results of the regression analysis between the recovery coefficient (RC) and surface area-to-volume (SA:V) ratio for the infinity, 10-to-1, 5-to-1, and pooled target-to-background ratios of all kidney phantoms in the study

	Infinity TBR	10:1 TBR	5:1 TBR	Pooled
Equation of best fit	RC [%] = 99.8 − 16.1 * SA:V [cm^−1^]	RC [%] = 99.8 − 16.1 * SA:V [cm^−1^]	RC [%] = 103.0 − 15.6 * SA:V [cm^−1^]	RC [%] = 100.9 − 16.0 * SA:V [cm^−1^]
*R*^2^	0.96	0.97	0.98	0.95

## Discussion

### Phantom Properties

Visually, the phantoms obtained in this study reproduced the structure of the patient’s kidneys exceptionally well. The phantoms were fillable and watertight after post-processing, thus enabling the determination of the extent of PVE as occurring in the individual patient’s geometry. However, the background compartment used and the position of the kidney phantoms in this compartment were not patient-specific; thus, fractions of scattered and attenuated photons of kidneys probably differed between patients and respective phantoms, which could be deemed a study limitation. Nevertheless, we believe that the attenuation and scatter correction of images using SPECT would compensate for these potential limitations so that we expect no major changes in RCs between our phantoms and in vivo.

Our phantoms had a wall of approximately 1.2 mm in thickness around the actual fillable volume, which might also be considered a study limitation. However, the wall is considered an inactive structure and is commonly termed a “cold wall” in the literature. Although we did not evaluate cold wall effects on RCs, Berthon et al. measured RCs for two different wall thicknesses (about 1.5 mm and 0.18 mm) and different TBRs [13] using similar PET scans of different-sized fillable spheres and found that RC bias was greatest at low TBRs and for smaller spheres. They also found a thicker wall resulted in lower RCs at a TBR of 4.8:1; the RCs were 5% lower than those for thin walls. At maximum, the RC was 8% lower for the 14 mL sphere. By comparison, our lowest TBR of 5:1 was similar to that of Berthon et al., but our phantoms were larger, and the two other TBRs in our study were higher. Therefore, even when considering the lower spatial resolution of SPECT compared with PET, we expect that only minor RC bias would have been produced by the cold wall effect, and, intuitively, a cold wall would have no effect on the RCs for the infinity TBR (cold background).

It has to be mentioned that our choice of parameters could influence the size of surface area of organs. If the spatial resolution of CT images increases (resolving smaller details), the details in surface-defining features increases, potentially increasing the measured surface area as well. This effect is similar to length measurements of coastlines based on maps resolving smaller details. Thus, the scanning and reconstruction parameters of the CT images used for segmentation could affect the measured surface area, which should be taken into account. For generating the phantoms and determining surface area and volume, we chose the degree of smoothing in the CAD processing so that the spatial resolution of CT images was of minor importance. Nevertheless, one should keep in mind that the regression between RC and SA:V could depend on the level of detail of segmented images and also on the degree of smoothing during further processing (if applied). The regression results obtained are thus specific for a given imaging and processing setup. Clearly, also the implementation of smoothing in our CAD program is proprietary, other programs could potentially lead to different results.

### Extent of the PVE

The RCs determined in phantoms reconstructed with the two different iteration numbers 36 and 72 differed significantly; however, their difference was only minor (1.2 percentage points, pps). Due to the nonlinear convergence of the iterative reconstruction method used in this study, we believe that higher iteration numbers than 72 would probably change RCs only marginally. Furthermore, our iteration number is representative of that used in a typical clinical setting. It is important to note that all herein reported results were obtained for one radionuclide and one specific SPECT camera. Other choices for, e.g. radionuclide, SPECT camera, and collimators could potentially change the results.

As expected, the 5:1 TBR RCs were significantly higher than the 10:1 and infinity TBR RCs by about 4 pp for all phantom types. This is most likely due to activity spill-in from the background, partially compensating spill-out activity from the kidney compartment. RC differences between the 10:1 and infinity TBRs were not statistically different. We chose our set of TBRs to maintain consistency with other studies that also applied infinity [14], 10:1 [5], and 5:1 TBRs [13]. Since differences between the two highest TBRs were insignificant, we did not perform experiments using a TBR between infinity and 10:1. However, the effect of lower TBRs, for example of 2:1, could be evaluated in future studies to cover a broader range of contrast ratios.

Typical RCs for ellipsoid, whole-parenchyma, and cortex-only phantoms were, respectively, 85.4%, 77.1%, and 49.1%, using the infinity TBRs and 72 iterations. Other TBRs and iteration numbers led to data within this range. We rated the observed RC differences between phantom types as substantial. At all TBRs, RCs for the cortex-only phantoms were significantly lower compared with other phantom types by about 27 pp. Most likely, this is due to increase in spill-out activity from the target to the background compartment as cortex-only phantoms with their finer structure are more affected by the PVE than whole-parenchyma phantoms. The boundary between high- and low-activity regions was larger for this phantom compared with the whole-parenchyma and ellipsoid phantoms. The intra-renal activity distribution influences the extent of PVE significantly. All applications relying on SPECT-based quantification of kidney activity in clinical practice are supposed to benefit from PVE corrections taking the intra-renal distribution of the radiopharmaceutical into account. For example, potential differences in the intra-renal distribution of [^177^Lu]Lu-DOTA-octreotide and [^177^Lu]Lu-prostate-specific-membrane-antigen could lead to differences in PVE and subsequent differences in dosimetry when these differences are neglected. Depending on the direction of bias, one could potentially under- or overestimate the associated radiation risk. For example, in a similar study, Tran-Gia et al. designed two compartment phantoms (renal medulla/collecting system and cortex), mimicking an anthropomorphic kidney based on a simple geometric model and printed these using a 3D printer [14]. Using SPECT/CT measurements with an identical scanner as in our study (Siemens Intevo Bold), but with ^177^Lu, medium-energy collimation and slightly different reconstruction parameters (48 vs 72 iterations in our study), they found an RC of 60.2% for the cortex-only phantom at an infinity TBR. This is higher than the RC of 49.8% (infinity TBR, 72 iterations) seen in our study, although our SPECT/CT scan has a higher spatial resolution because we used ^99m^Tc and LEHR, theoretically resulting in higher RCs and lower PVE. These authors also reported an RC of 82.0% for the ellipsoidal phantom, which was similar to our result of 85.4%, especially when we account for the higher spatial resolution of the ^99m^Tc-SPECT scan. We suspect that the difference in RCs for the cortex-only phantom between their study and our study was probably due to geometric differences of the phantoms used: Our phantoms more closely reflected patient-specific geometry than the phantom of Tran-Gia et al. that had a more simplified geometry. For our phantoms, the surface area-to-volume ratio is probably higher, which significantly correlates with the extent of PVE and thus the RC, as seen in Fig. 5 and discussed herein.

Of note was the higher inter-phantom/inter-patient RC variability of the cortex-only geometry compared with the whole-parenchyma geometry, which potentially limits the efficacy of PVE corrections based on phantom measurements or computational simulations of average patient geometries, as indicated by the 38.0% to 60.6% RC range for the infinity TBR and 72 iterations. If this bias could be corrected using the average cortex-only phantom RC of 49.8%, a maximum under- or overestimation by -11.8% and 10.8%, respectively, would occur.

### Regression analysis between surface area over the volume and recovery coefficient

PVE measurements are typically carried out with simple phantom geometries such as spheres. Consequently, correcting tumour PVE with RCs derived from these spheres is recommended [2]. Spherical phantoms are readily available and might be useful when PVE-correcting uptake values measured in structures close to their geometry such as small pulmonary nodules; however, for organs of higher geometrical complexity such as the kidney they would be expected to be of limited value. Furthermore, identical object volumes will not guarantee identical RCs, even if the TBR and spatial resolution of the imaging system are constant, as already indicated by prior studies [14]. The RCs of compact objects, such as spheres or ellipsoids, are not identical to the RCs of more complex, organic structures, as shown in our study. Clearly, the size of the interface between hot (more active) and cold (less active) structures influences the extent of PVE. Intuitively, we expected the RCs to be smaller if the surface area (SA) of the object (and thus interface size between hot and cold regions) increases, suggesting a negative correlation between the RCs and SAs. On the other hand, the RC is higher for larger (higher volume) objects, suggesting a direct positive correlation between the RC and volume. Combining these results, the SA:V ratios correlated well with the RCs and TBRs in our study. For example, the linear regression analysis for the pooled TBR yielded an *R*^2^ = 0.95 (Fig. 5). The width of the 95% prediction interval indicates that the extent of PVE could reasonably be estimated within ± 6.8% RC, based on the SA:V ratio. We expect that calculating SA:V ratios would be easier to perform than simulating the imaging process using phantoms with subsequent RC determinations. However, an important limitation of our model is that the linear regression performance did not account for differences in the spatial resolution of the imaging system. Moreover, we have only studied objects with shapes similar to a kidney. Future studies could focus on a more complete modelling of the PVE. In this context, it has to be mentioned that Amato et al. [15] Introduced the generalized radius r’ = 3*V:SA, which is essentially the reciprocal of our measured SA:V. We decided to not implement the generalized radius, since this would change the regression with PVE from a linear to a hyperbolic type, which would be less intuitive and could potentially impact the application of the PVE correction in clinical practice.

### Potential clinical benefit

Despite the limitations outlined above, our study provides an estimate of the magnitude of the PVE in individual patients, allowing for better modelling of errors in dosimetry. In addition, our approach to create patient-specific phantoms could be used for individualizing doses of radionuclide therapies, in which the kidney is the critical organ. Highly desirable for our approach would be knowledge on the distribution of the radiopharmaceutical in the different compartments of the kidney, as the differences in RCs demonstrated between the whole-parenchyma and renal cortex phantoms indicates. Clearly, studies comparing the results from the different dosimetric approaches with biological effects in a clinical setting might help determine their clinical value.

Furthermore, we also see a significant potential of our phantoms for evaluating techniques to correct PVE. The SA:V ratio is a metric that could be measured in clinical practice and could be used directly as a first approximation to correct PVE, improving dosimetry results and potentially leading to improvement in estimating the risk of side effects in patients receiving ^177^Lu therapy. Theoretically, all methods for determining surface area and volumes of a patient’s kidneys as outlined herein could be applied in clinical practice, although the availability of the CAD software necessary for this could be challenging. Finally, yet importantly, future experiments and simulation studies addressing other radionuclides, SPECT systems, and organs are necessary to establish the validity of our approach to use phantoms tailored to the individual patient’s anatomy for PVE correction. Additionally, the question remains whether the surface-area and volume are sufficient for estimating recovery coefficients for other geometries or if other measures for shape and size would be needed.

## Conclusions

We have shown that changes in patient-specific kidney-shaped phantoms cause large changes in the ability to determine the extent of PVE. Therefore, RCs derived from more simple phantoms are most likely insufficient for the PVE correction of patient images. Furthermore, one should account for the distribution of intra-renal activity that significantly influences the extent of PVE. Also, we demonstrated that the SA:V ratio is an excellent tool to model the RCs suited to PVE-correct images of tracer uptake within the kidneys. Potentially, this ratio could also be applied to other organs and could be used as an alternative to the full simulation of imaging processes that are currently performed to determine the extent of PVE.

## Data Availability

The datasets used and/or analysed during the current study are available from the corresponding author on reasonable request.

## Abbreviations

SPECT: Single-photon emission computed tomography
PVE: Partial volume effect
RC: Recovery coefficient
SAV: Surface area-to-volume
PET: Positron Emission Tomography
VOIs: Volumes of interest
FWHM: Full width at half-maximum
MRI: Magnetic resonance images
CAD: Computer-aided-design
FDM: Fused deposition modelling
PLA: Polylactic acid
PVA: Polyvinyl alcohol
TBRs: Target-to-background ratios
HU: Hounsfield
LEHR: Low energy high resolution
MELP: Medium energy low penetration
OSCG: Ordered-subset conjugate-gradient
pp: Percentage points
SD: Standard deviation
SA: Surface area
